# Recanting Children’s Descriptions of Influences and Pressures to Recant Intrafamilial Child Sexual Abuse

**DOI:** 10.1177/10775595251408453

**Published:** 2026-01-02

**Authors:** Zsofia A. Szojka, Breanne E. Wylie, Agnieszka M. Nogalska, Annabel Reay, Thomas D. Lyon

**Affiliations:** 1 4918 School of Law and Criminology, University of Greenwich, London, UK; 2 Department of Psychology, North Carolina State University, Raleigh, North Carolina, USA; 3 Feinberg School of Medicine, Northwestern University, Evanston, Illinois, USA; 4 Department of Psychology, University of California, Irvine, California, USA; 5 Gould School of Law, University of Southern California, Los Angeles, California, USA

**Keywords:** recantation, child sexual abuse, intrafamilial abuse, forensic interview

## Abstract

This study examined interviews with 53 4 to 15-year-old children recanting sexual abuse to determine whether they were forthcoming about potential influences and pressures that could have led them to recant. The great majority (87%) of children mentioned one or more influences or pressures. With respect to influences, about half of children mentioned their positive feelings for the suspect, and about a third mentioned negative consequences for the suspect. About 30% disclosed that their immediate family missed the suspect. About half mentioned concerns about separation from their family, and almost 30% discussed negative consequences for their mother. Pressures from others were mentioned by about half of children. They rarely mentioned pressures from the suspect, but over a third disclosed family pressures, most often the lack of maternal support. Over two-thirds of children described feeling guilty and other internal influences. The results provide preliminary support for questioning recanting children about influences and pressures.

## Introduction

An essential issue in allegations of child sexual abuse is whether the child’s report is true. Although disclosure of abuse obviously increases the likelihood that abuse has occurred, investigators must consider whether the disclosure is false. Concerns about false disclosures frequently involve allegations that the child’s report was influenced in some way by adults, usually caretakers or professionals who falsely believed that abuse occurred or were deliberately pressuring the child to create a false allegation (Principe & London, 2022). In some cases, children recant their initial allegations. Under normal circumstances, recantations decrease the likelihood that abuse occurred, but as with original allegations, investigators must consider if the recantation is false (Bücken et al., 2022; Korkman et al., 2024; Lyon et al., 2026; Talwar & Crossman, 2025).

Research examining the relation between case characteristics and recantation suggests that children are vulnerable to influences and pressures within their family that lead them to recant allegation of sexual abuse. They are more likely to recant when the suspect is a parent figure and the mother and other family members are unsupportive (Malloy et al., 2007, 2016). However, knowing the factors that are related to higher rates of recantation is of limited value in determining why children recant in individual cases (London et al., 2020). Children’s closeness to suspects will vary from case to case, and children often view caretakers’ support quite differently than the caretakers themselves (Bick et al., 2014; Morrison & Clavenna-Valleroy, 1998). Children’s perceptions are key.

An unexplored issue is whether children who recant during a forensic interview might be forthcoming about influences and pressures to recant. This would support questioning recanting children in order to understand their reasons for recanting. This study examined 53 interviews with 4 to 15-year-old children recanting allegations of sexual abuse and measured the extent to which they disclosed influences and pressures which could explain the recantation. Influences could be both external and internal: External influences refer to potential reasons for recantation resulting from the child’s relationship with and feelings about other people, and internal influences refer to more general statements about the child’s cognitions and emotions. Pressures refer to direct expressions of disapproval of the allegation or attempts to persuade the child to recant. In what follows we review prior research examining the factors that increase the likelihood of recantation and discuss the legal significance of recantations, including how the courts view the significance of influences and pressures to recant.

### Correlates of Recantation

Malloy et al. (Malloy et al., 2007, 2016) proposed a filial dependency model of recantation, whereby the likelihood of recantation is increased by factors that reflect the child’s dependency on their family and their susceptibility to influences and pressures. Examining sexual abuse cases substantiated by social services investigation, Malloy et al. (2007, 2016) found increased rates of recantation among children alleging abuse by a parent figure, and in cases with unsupportive caretakers (typically mothers) and family members. Other research has similarly found higher rates of recantation when the suspects were parent figures (Baía et al., 2021), family members (Celik et al., 2018), or familiar to the child (Hershkowitz et al., 2007). Other research has also found that recantation rates are higher when the child doesn’t receive support from mothers (Elliott & Briere, 1994) and family members (Baia et al., 2021). These factors mirror reasons why children never disclose, delay disclosing, and deny abuse when first questioned. Research examining non-disclosure, delayed disclosure, and denial have found that closeness to the suspect, maternal support, and familial support decrease the likelihood that children disclose (Lyon et al., 2026).

With respect to closeness to the suspect, several reviews of the literature have noted that participants who report having failed to disclose abuse attributed their failure to positive feelings for the suspect and fears concerning the negative effects of disclosure for the suspect (Lemaigre et al., 2017; Morrison et al., 2018). In a population survey questioning respondents who reported past sexual abuse to the surveyor but had failed to disclose, Anderson et al. (1993) found that a primary reason for non-disclosure was a wish to protect the suspect. Conversely, suspects close to the child are also in a better position to punish the child for disclosure, and both the population survey by Anderson et al. (1993) and a review of the literature (Alaggia et al., 2019) identified fears of the suspect and concerns about the child’s own safety as reasons for non-disclosure.

With respect to reactions of the non-offending caregiver (typically the mother) and the family, studies examining barriers to the disclosure of child sexual abuse found that children were more likely to delay disclosure when they feared the consequences of the disclosure to others (Goodman-Brown et al., 2003) or anticipated a negative response from caregivers (Hershkowitz et al., 2007; see Alaggia et al., 2019 for a review). Supportiveness is typically measured by assessing whether the caretaker believes the child, provides emotional support, and takes steps to protect the child from the suspect (Cyr et al., 2003). Substantial percentages of caretakers fail to act supportively when abuse is alleged. Comparing rates across studies asking mothers about their supportiveness, Cyr and colleagues (2014) noted that whereas 65 to 78% of mothers reported that they believed their children, half provided emotional support, and half quickly took action to distance their child from the suspect. Hence, up to a third of mothers surveyed didn’t believe their children and half failed to provide emotional support or protect the child. Caregiver support is often closely related to the identity of the suspect. When the child accuses a family member of abuse, mothers may feel torn between their child and the suspect, especially if the suspect is the mother’s romantic partner. Indeed, maternal supportiveness is negatively related to whether the mother is cohabiting or in an intimate relation with the suspect (Bolen & Lamb, 2002; Cyr et al., 2002, 2003; Everson et al., 1989; Walsh et al., 2012).

An alternative explanation for the relations among recantations, suspect-child relationship, and maternal supportiveness is that recantations demonstrate that the original allegations were false, and false allegations are more likely when the child is close to the suspect and the mother doesn’t believe the allegation. However, Malloy et al. (2007, 2016) limited their sample to cases in which abuse had been substantiated by social services. Moreover, when examining cases with corroborative evidence, Malloy et al. (2007) found non-significantly lower rates of recantation (see also Baia et al., 2021; Elliott & Briere, 1994; who focused on cases with corroborative evidence). Furthermore, it is in cases in which the mother believes the allegation, and aggressively pursues it, when suspicions are greatest that the child’s original allegation was false (Principe & London, 2022). Hence an unsupportive mother reduces concerns that she has coached or influenced the child to make a false accusation.

### Legal Significance of Recantation

Because investigators and factfinders rely heavily on children’s disclosures in order to determine if abuse has occurred, recantations are likely to lead to a conclusion that abuse did not occur or could not be proven. Large percentages of children recant when they are first formally questioned about abuse (Leach et al., 2017; Lippert et al., 2009), and social services are unlikely to substantiate an abuse allegation without a formal disclosure (Haskett et al., 1995). Similarly, researchers will often presume that these cases reflect false allegations because of the possibility that the original disclosure could be the result of pressure or misinterpretation by an informal recipient (London et al., 2005). In criminal cases, recantations and other inconsistencies in the child’s report reduce the likelihood that the police will present the case for prosecution (Davis et al., 1999; Stroud et al., 2000), make it less likely that the prosecutor will file criminal charges (Bracewell, 2018; Walsh et al., 2010), and if charges are filed, increase the likelihood that the case will be dismissed (Christensen et al., 2014).

Although recantations typically undermine the legal substantiation of abuse, there is some recognition among legal professionals that recantations can be the result of influence or pressure on children to change their story. Experts frequently testify that recantations are an understandable reaction to influences and pressures (*California v. Munch*, 2020; Horan & Goodman-Delahunty, 2020), though experts for the defense will argue that recantations of true allegations are uncommon (*Ohio v Swoboda*, 2021). Dependency courts, which require a lower standard of proof than criminal cases, sometimes find that abuse occurred in spite of recantations, even when the child recants under oath on the stand (*In re E.G.*, 2025).

If a child recants after testifying in a criminal case that abuse occurred, “recantations are viewed with extreme suspicion” (*Case v. Hatch*, 2013, p. 1041). In assessing the credibility of the recanting child, courts will assess whether there is evidence of “collusion between the defendant and the witness” or “undue influence or pressure from any source” (*New Mexico v. Worley*, 2020, p. 1221). More subtle influences are often also recognized, such as whether “the complainant recanted after moving in with family members of the defendant” (*Keeter v. Texas*, 2002, p. 38). On the other hand, if there is evidence of pressure to make a false allegation, and little corroborative evidence, recantations are often a basis for reversing convictions (Klassen et al., 2025).

### Prior Research on Questioning Recanting Children

Research specifically examining recantation, as opposed to non-disclosure and delayed disclosure, has exclusively focused on factors that can be externally measured rather than inquiries into what children report, including whether internal influences (such as guilt and fear) played a role in their decision to recant. We are aware of only one study examining forensic interviews in which children recanted. Katz (2014) examined 12 cases in which 5- to 13-year-old children recanted allegations of physical or sexual abuse during an interview. In all cases children had requested the interview, and all “explained their reasons for recanting” (p. 162). Although Katz did not formally classify influences and pressures, children referred to rejection by the suspect (“he does not want me anymore,” p. 162); rejection by the mother (“my mom does not want me anymore,” id.), separation from the mother (“I just want to go home to my mom,” id.), and negative consequences for the family (“my words can destroy my whole family,”; “my grandma will die [without the suspect], id.). Katz’s findings (2014) suggest that interviews with recanting children may be a promising source of information about influences and pressures.

### The Present Study

The purpose of this study was to examine recanting children’s description of the influences and pressures they experienced following their allegations of child sexual abuse. Thematic analysis was used to identify, analyze and report themes in 53 forensic interviews with 4- to 15-year-old children. We calculated the percentage of cases in which children mentioned different influences and pressures and identified direct quotations in individual interviews that illustrate how children talk about potential reasons for recantation.

## Method

### Sample

We examined 53 forensic interviews with 4- to 15-year-old children (*M* = 10.92, *SD* = 2.66; 91% female) who recanted allegations of sexual abuse during a forensic interview. Recantation was defined as complete denial that any sexual abuse had taken place following a disclosure of abuse. In all but four cases (93%; *n* = 50) the sexual abuse allegations were substantiated by the Department of Children and Family Services (DCFS) investigation and a dependency petition was filed for the dependency court to take jurisdiction over the child. In three cases DCFS had closed their case following the recantation, either classifying the case as “inconclusive,” (69, 129) which refers to insufficient evidence to make a determination, or “unfounded,” in which the investigator concluded that abuse did not occur (321; Cal. Penal Code Section 11165.12, 2025), and in one case the disposition was unknown (585). (Numbers refer to our archived database of forensic interviews.) We were able to obtain dependency court records in 90% (*n* = 45) of the substantiated cases, and in the remaining five cases we were able to determine that DCFS had substantiated the case based on the child’s disclosure (e.g., reference to court appearances or speaking with their attorney; 107, 123, 215, 440, 446).

The interviews were conducted between 11/20/2007 and 11/17/2021 by forensic interviewers at nine Child Advocacy Centers in Los Angeles County. Cases were referred to the Child Advocacy Centers by law enforcement or child protective services for possible criminal prosecution and dependency intervention. Dependency intervention occurs when it is necessary to interfere with a parent’s (or parents’) custodial rights in order to protect children from maltreatment. The interviewers would have received training in the 10-Step Protocol (Lyon, 2005, 2021), a revision of the original NICHD Protocol. The interviews were transcribed and anonymized for training purposes, either with the consent of the parents/guardians or, when the children were under the jurisdiction of the dependency court, with the consent of the court. The use of archived interviews for research purposes was approved by the University of Southern California Institutional Review Board as exempt (45 CFR Section 46.014(d) (4) (ii)).

Dependency court records or Child Advocacy Center intake forms with information about the alleged perpetrator and type of abuse were available for 48 (91%) cases. All cases involved allegations against a family member. Eighty-four percent of victims alleged that they were abused by a father figure, with the suspect named as the father in 29% of all cases and the stepfather in 54%. The remaining allegations involved siblings (10%), grandfathers (2%) or other family members (4%). Four children (8%) had originally disclosed abuse by multiple suspects (father and family friend, stepfather and family friend, stepfather and unspecified suspect, sibling and cousin).

Forty-two percent of children described multiple types of sexual acts involving the suspect; 58% described a single type of sexual act. Twenty-five percent of cases involved penile penetration of the child, 10% digital penetration of the child, 15% oral copulation of the child, 10% oral copulation of the suspect, 23% fondling of the child under clothes, 2% fondling of the suspect under clothes, 56% fondling of the child over the clothes and 8% fondling of the suspect over clothes.

### Coding and Analysis

In order to categorize children’s statements, we defined external influences as potential reasons for recantation emanating from the child’s relationship with and feelings about other people, pressures as statements and actions by others demonstrating disapproval of the allegation, and internal influences as the child’s emotions and cognitions that could motivate recantation. Broad initial themes (suspect-related influences, family-related influences, suspect pressures, family pressures) were identified based on a preliminary read of a subset of cases by all five authors. Next, two independent coders examined all 53 transcripts using thematic analysis, and identified 19 distinct variables in seven main categories (see Table 1 for the list of variables). Thematic analysis is a qualitative research method used for identifying, analyzing, and reporting patterns (themes) within data (Braun & Clarke, 2006). It involves systematically coding data to capture important features and then organizing these codes into broader themes to interpret the data’s meaning. The two coders independently coded every question-answer pair (*N* = 11,055) in all 53 transcripts for the presence or absence of each of the 19 variables. To support the interpretive agenda of thematic analysis (Braun & Clark, 2013) and emphasize reflexivity and active engagement with the data, disagreements between coders were resolved through conversations involving all five authors instead of relying on quantitative measures of inter-rater reliability. For purposes of quotation, pseudonyms were assigned to children such that a child’s pseudonym matched the child’s gender and ethnicity based on Tzioumis’ (2018) calculation of the most common first names among different ethnic groups. Quotations in the results section were edited for clarity and redundancy.

**Table 1.** Influences and Pressures Disclosed by Children in Their Forensic Interview

Influence or pressure	% Cases	*n* cases
**Any external influence**	**87**	**46**
*Suspect-related influences*	*68*	*36*
Positive feelings for the suspect	51	27
Negative consequences for the suspect	34	18
Siblings miss the suspect	32	17
Mother misses the suspect	28	15
Positive contact with the suspect	30	16
*Family-related influences*	*74*	*39*
Separation from family	55	29
Negative consequences for the mother	28	15
Economic support from the suspect	17	9
Negative consequences for the family	9	5
*Other influences*	*26*	*14*
Negative consequences for the child	25	13
Legal intervention	11	6
**Any pressure**	**49**	**26**
*Suspect pressures:* Coaching by the suspect	*4*	*1*
*Family pressures*	*38*	*20*
Lack of support from the mother	26	14
Lack of support from the family	17	9
Coaching by the mother	11	6
*Other pressures*: Lack of support from professionals	*6*	*3*
**Internal influences**	**70**	**37**
Guilt feelings	53	28
Failure to anticipate consequences of disclosure	40	21
Negative emotions	38	20
**No influence (external or internal) or pressure disclosed by child**	**13**	**7**

Bold values represent any external or internal influences or pressures. Italic values represent broad themes within influences or pressures.

## Results

### External Influences to Recant

Almost ninety percent of children (87%; *n* = 46) disclosed one or more influences or pressures (Table 1). Among those children, all described at least one type of external influence, with most of the influences related either to the suspect (68%) or the child’s family (74%).

**Suspect-Related Influences** Influences related to the suspect were disclosed by almost two-thirds of children (68%). About half of children (51%) described their ***positive feelings for the suspect*.** Forty-six percent of children reported general positive feelings for the suspect, and 24% explicitly said that they miss the suspect. Many children expressed their wish for the suspect to come back to the family home.

I just wanna talk to my dad or send him cards or anything or things I draw to my dad, send photos, and I love my dad and I want my dad to know that I still care for him and that I still want him to live with us. (Sidney, 12, 1139)

Q: How come you’re feeling sad? A: It’s just that I miss my dad. (Madison, 9, 1154)

Children took pains to emphasize the care and support they received from the suspect and highlight his moral character.

I felt really bad… cause he’s a good guy and like he respects us and we respect him, and then, he treats us like if were his daughters, he buys us everything we need… and I felt bad that I said that story because…he wouldn't do anything like that to us. (Amanda, 11, 446)

I just feel sad, ‘cause my brother was a good boy. He was a nice and kindful person, and he’ll buy a lot of gifts on Christmas for people and… he’ll sometimes give money too when they need it, and… give good advice and he was the person that everyone really liked. (Emily, 12, 1317)

Over a third (34%) of children reported ***concerns about potential negative consequences for the suspect***.

I was thinking about what would happen to my dad…if they were to arrest him. I was thinking he’ll probably get injured over there, they’ll probably hurt him over there, they are not gonna take care of him right. (Sidney, 12, 1139)

Just feeling guilty about it, like I could’ve put him in jail or made him go back to Mexico or something. He has his papers and everything but it’s just that they could’ve done that to him. And also ruin his record… he can’t get his job, he won’t be able to. (Valentina, 12, 1093)

Thirty-two percent of children reported that ***their siblings miss the suspect***.

[T]hey say that they miss their dad, but you know, I just tell them that… hopefully one day they’ll see him. (Zara, 13, 1279)

[L]ike for my baby brother since my stepdad is the biological father, he got really affected and since he’s not able to see him, he’s able to see him at phone calls or FaceTime, to see him not face to face. And… he cries more, he’s changed a lot. (Gabriela, 12, 1379)

Twenty-eight percent of children reported that ***their mother misses the suspect.***

Sometimes she tells me she doesn’t want to be alone anymore, and she just wants to be with like someone that she loves which is their [child’s siblings’] dad. (Daniela, 14, 129)

She would cry a lot and finally after like two days after he got arrested she finally told me how they met and about my biological father. I knew he was dead but I never found out why,… so she finally told me the whole story and it was really sad and then she told me that he was always there and he said that he wanted to finish what my biological father couldn’t finish. And then I thought about our future without him, like not money-wise but just like when I’m gone and my brother’s gone and like we’re like having our own lives and she’s gonna be all lonely. (Valentina, 12, 1093)

Thirty percent of children reported continuing ***positive contact with the suspect***.

[H]e said that we’re gonna try and fix things and that I'm not alone in this, and that he’s not mad at me…. Like I guess he was just trying to comfort the whole family and me. (Olivia, 14, 1186)

**Family-Related Influences** Influences related to the child’s family were disclosed by 74% of children. The most common theme focused on ***separation from their family*** or wanting to be reunited with their loved ones (55%).

Q: So what do you want to happen now? A: That my family comes back and my dad and my mom come back with me and have some fun, like a big family. (Laura, 9, 932)

I wanna go with my mom again. They took me away too ‘cause I said that. (Elijah, 10, 1099)

Children often reported that they started to regret having made the allegations when the possibility of being removed from home was brought up by the police or social workers.

Q: And what were you thinking when you told the police? A: I like kind of like regretted cause they like brought up foster home. (Camila, 15, 1030)

A: I didn’t like [the social worker], I’m sorry. She was the one that, because of her, I felt pressure ‘cause she’s like, you’re never gonna see your mom again, and you’re never gonna get back to her… So I just had like my anxiety got worse since then, like I have really bad anxiety now, even worse than what I had.. (Morgan, 13, 1108)

Children who were removed from their home sometimes described being taken to foster care as a confusing and traumatic experience.

[W]hen they told me to go with them, I’m like, where are you guys taking me? And they said, we can't tell you where. But after that they took me to a foster place and I'm like, this is not my sister's house. And I told them, why are you guys taking me? We couldn't tell you, but this is a place where you can stay safe. And I told them, I don't want to go to foster care, ok? I want to go to my sister or my mom. (Lauren, 12, 123)

Children also mentioned being worried about the ***negative consequences for their mother*** (28%). Caregivers’ initial reaction to the allegations was often negative, and even when their mother was supportive, children sometimes felt guilty about the emotional distress caused by the allegations.

Q: What did your mom do after you told her about the touching? A: Well, she was just sad. She was laying down on the bed, sad all alone in the dark. So I actually felt bad for my mom so yeah and I walk away thinking that I was gonna cry. (Victoria, 10, 1129)

Caregivers’ emotional distress profoundly influenced children, with twelve-year-old Gabriela (1379) explicitly stating that she recanted her allegations just a few days after she told her mother about sexual abuse by her stepfather because she “didn’t want her suffering.”

Beyond the emotional impact of the allegations on their caregiver, 17% of children also disclosed the practical consequences of the suspect leaving the home, such as the ***family losing economic support.***

Q: Why would losing your stepdad scare your mom? A: Because she can’t afford the rent… (Jasmine, 13, 585)

He pays our phones, sometimes when my mom asks him money for like asks him, like calls him and asks him for money for us. (Caroline, 11, 1454)

Nine percent of children also described ***negative consequences for their family*** more broadly, mentioning others in the family besides their mother.

I saw my mom hurt… my family was hurt about it,… my aunt that lives with us was hurt,… and I was starting to regret saying anything about it being true or saying that he did something to me…. (Jasmine, 13, 585)

**Other influences to Recant.** A fourth (25%) of victims mentioned ***other negative consequences*** they experienced as a result of their allegations, such as missing school due to the investigation and concerns about schoolmates learning about the allegations.

Q: And how has… the big case been for you? A: Stressful, difficult, cause I keep missing days of school and I don't want to. And… I'm missing like some assignments… and that I have to do a bunch. Instead of doing them, I can't, cause it's either court or go to this place and go to that place and it really does get boring cause like I just wanna like do my stuff already and get like all my credits like completed so I can go to junior year next year. (Camila, 15, 1030)

[A] lot of kids at school saw the cops and they were like, oh did you see the cops outside, and I got scared, I was like hopefully they don’t find out what happened ‘cause… they end up spreading it all around the school and like everybody will find out about what happened. (Sidney, 12, 1139)

Eleven percent of children explicitly noted that they opposed ***legal intervention***. Some children felt overwhelmed by the investigation and felt frustrated about having to retell their story repeatedly.

I thought that it was too much because…you have the police involved and going to court, and… I’m like, no. Well the situation is going out of hand. (Fatima, 12, 1102)

It’s just that I don’t like talking to people. I know I don’t like people talking to me. Now it’s making me sad [child cries]. (Madison, 9, 1154)

### Pressures to Recant

Pressures were mentioned by about half of children (49%). These included suspect pressures (4%), family pressures (38%), and pressures by professionals (6%).

**Suspect Pressures.** In contrast to influences from the suspect, overt pressures from the suspect were rarely mentioned, in 4% of cases. Direct coaching by the suspect to withdraw the allegations was discussed by only one child, 12-year-old Jennifer (992), whose grandfather, the alleged suspect of the abuse, instructed her to tell her teacher that she lied about the abuse.

[M]y mom told me that I wasn't, like the social worker said that I wasn't able, I wasn't able to be contacted by my grandpa. I wasn't able to contact him or call him or see him. I did something wrong at school. I asked my friend if I could use her phone to call my grandpa and she said she'd let me, so I have his number memorized. I called him and he wasn't angry. He was just happy to like, to hear my voice and he just told me to tell anyone from my school… that it was a lie, and that to do the correct thing cause he said saying more lies will make it even worse. So I did what he told me to do, I went to my art teacher. (Jennifer, 12, 992)

**Family Pressures.** 26% of children said ***that their mother was unsupportive*** of the allegations. Children reported that their mother did not believe the allegations because they found the child’s story implausible and trusted the suspect.

And… that’s when I talked to… the principal and then that’s when my mom came and she told him the real story that it wasn’t true ‘cause… my little sisters and brothers are at the house and he wouldn’t ever do that. And the same day I wasn’t home by myself. (Daniela, 14, 129)

Children often disclosed that their mother repeatedly questioned them about the allegations.

She said… if I was lying… and I knew that she didn't believe me. And she was like please tell me the truth. I was like mom, I am telling you the truth…. She would vomit a lot during nighttime. She would get up and she'll vomit and she would think about and cry a lot. She was really desperate and right here it will be black all the time. And that's when I felt sorry, so I just said I'm sorry like it was a lie. And she was crying more, but she was mad also. (Nevaeh, 12, 937)

It was just me, my mom, and my siblings, and you know, my mom kept telling me, like “Is it true that he did that?”....[Later s]he was explaining… to her friend… how it happened and what happened, and then you know she looked at me and she was like, and she’s like “Tell me the truth. Did it, did he actually do it or did he not?” (Zara, 13, 1279)

Mothers sometimes expressed frustration with the child in more subtle ways.

She’s pretty sad, she like, the thing that annoys me the most and gets me mad is that when I’m around, it’s literally when I’m around she always talks about [the suspect], making me regret even thinking about getting revenge and getting him out of the house. (Lucia, 13, 1459)

He can’t be near us, we can’t go near him, we can’t see each other, we can’t talk to each other, and all that. And then my sister’s like, “where’s daddy? I miss him. Where is he? I miss him,” then my mom just gave me like a look, like a dirty one, like mean. (Jasmine, 13, 585)

Seventeen percent of children reported that someone in their wider ***family was unsupportive of the allegations***. Siblings often explicitly accused the child of lying, whereas other family members expressed shock and questioned the child repeatedly about whether the allegations were true. In addition to being repeatedly questioned by her mother, thirteen-year-old Zara was questioned by her mother’s friend, and later by her Godmother, to whom she finally recanted.

[M]y god mom said she wanted to talk to me, and I was like, ok. So, then we sat down and…she was almost in tears already…[S]he calls my stepdad, Pa… and so she’s like I’ve seen how Pa acts around you… He acts like any normal father would…he buys you things. He gives you the things you need, and he helps your mom and makes your family happy…I’ve seen how you are with him. You guys are so…happy around each other… You guys never seem any different than you guys had since I’ve been here… but if he really did that to you…then I guess you keep telling the story, but…I feel like there’s something that’s not right…. I feel like when you say that it’s true, you look like, you know, you look like you’re holding back something…. I just looked at her for a little bit, and I told her he did. And then she was like, he did what? And I stood quiet and then she’s like, why, you know, if it was true you need to tell me without looking around or, you know, stuttering ….And then I just stood quiet for a little bit, and I remember she hugged me, and she was like crying, and then she’s like, did he do it or did he not? (Zara, 13, 1279)

[M]y sister Paige, she said I can't believe you did that cause she said she didn't really like the guy, but that's like the worst thing to lie about. (Amanda, 11, 446)

Eleven percent of children disclosed ***coaching by the mother***. Coaching usually involved telling children not to say anything to the authorities.

I wanted to ask the police but my mom say that don't tell them nothing. Just be quiet and then I say ok mom. Then I want to say it but she say no, don't say it. (Cora, 7, 893)

Some mothers attempted to convince their child that they misinterpreted innocuous touch as sexual.

She told me…there are two like different things with sexual and non-sexual. Like this was sexual, but the patting [pats thigh] wasn't sexual. And then if… a guy puts his head on your chest, then that's sexual. If he puts his head on your shoulder, then that's not sexual. (Camila, 15, 1030)

**Professional Pressures.** In six percent of the cases children said that professionals were unsupportive of the allegations.

[The detective] showed the papers that they spent money on. And he said there's no possible way that your dad could have done this. And I said I know there wasn't. (Cassidy, 13, 851)

The doctor just took a look and told my mom no one has touched me, and that it might be the growth of hair that’s growing in my middle part… or the soap that I’m using is causing the itchiness. (Fatima, 12, 1102)

### Internal Influences

More than half (53%) of children described ***guilt feelings***. Feelings of guilt were strongly associated with familial influences, with children describing feeling responsible for the negative consequences affecting their mother, their siblings and the suspect.

Q: Mm-hmm. And how did that feel, getting taken away from your mom? A: I was scared and sad and I felt like it was my fault because I said all these things and that's what caused it. (Sarah, 12, 1036)

Q: I see, so how did that make you feel Gabriela, when you saw how your stepdad being gone was affecting your two brothers? A: Well, it was affecting me ‘cause I felt really bad and also because I didn’t want my little brothers to feel the same pain I went through when my biological father wasn’t there, or didn’t even take care of us… (Gabriela, 12, 1379)

Many children (40%) failed to ***anticipate the consequences of disclosure***.

I didn’t know this was all gonna happen. (Jasmine, 13, 585)

I was like oh no, what did I do, what did I do. And I realized that I could get someone in big trouble for that and even myself in trouble for that. (Sarah, 12, 1036)

Apart from guilt, children often (38%) described other ***negative emotions***, including depression, loneliness, and in some cases thoughts of self-harm.

I feel like upset about that and really, really sad I am for all my family. (Laura, 9, 932)

[When she found out her friend had informed school officials of her disclosure] I wanted to go home and cut myself... Just felt like pain. (Jasmine, 13, 585)

Well sometimes I feel like committing suicide because I don’t get to see my dad anymore. I only get to see him one time, his visit. I’m gonna see him today, second time. And then when they told me I couldn’t see him, my heart fell apart. It’s like if an empty bottle was cracked. (Caroline, 11, 1454)

Internal influences often co-occurred with external influences in children’s reports. Thirty percent of children reported feelings of guilt and also mentioned concerns about the negative consequences of the allegations on their mother. Thirty-five percent reported that they did not anticipate the consequences of disclosure and also mentioned that the allegation resulted in separation from their families.

## Discussion

This study examined whether children recanting sexual abuse allegations in forensic interviews were forthcoming about influences or pressures that could have led them to recant. We reviewed the transcripts of interviews with 53 4- to 15-year-old children and found that almost 90% of children disclosed one or more influences or pressures. We categorized the influences and pressures with respect to the suspect, the child’s family, the child, and others (such as professionals). We also considered internal influences, as expressed by children’s references to their subjective feelings and beliefs.

Eighty-seven percent of children mentioned potential influences on their decision to recant. With respect to the suspect, about half of children mentioned their positive feelings for the suspect. About a third of children mentioned the negative consequences of their allegations for the suspect, about 30% disclosed that their siblings and their mother missed the suspect, and 30% described positive contact with the suspect. With respect to their family, children most often mentioned separation from the family (again about half of children). Almost 30% of children discussed negative consequences for the mother, and smaller percentages mentioned economic support from the suspect and negative consequences for the family. Finally, with respect to themselves, about a fourth of children mentioned other negative consequences, and a smaller percentage discussed their unhappiness with legal intervention.

About half of children mentioned pressures from others. However, only 4% of children mentioned suspect pressures, and only 6% mentioned unsupportive professionals. In contrast, over a third of children disclosed family pressures, most often referring to a lack of maternal support, lack of family support, and less often, overt coaching by the mother to recant. Over two-thirds of children mentioned internal influences, in which they described their subjective feelings. Guilt was most common, expressed by about half of children, with over a third of children mentioning their failure to anticipate the consequences of their allegation or other negative emotions. In what follows we discuss the implications of the findings for research on the dynamics of abuse disclosure and for forensic interviewers who question children. We then discuss the limitations of the study and directions for future research.

### Comparison to Prior Research

The findings are consistent with research identifying factors that increase the likelihood of recantation, including closeness with the suspect and a lack of support from the mother and other family members (Baía et al., 2021; Celik et al., 2018; Elliott & Briere, 1994; Malloy et al., 2007, 2016). The results are also novel in several respects. First, they demonstrate that recanting children can themselves be a source of information about influences and pressures, which will be particularly helpful when family members are unavailable, uncommunicative, or potentially dishonest about their beliefs and actions with respect to the allegations. Second, because interviewers can ask children about their subjective reactions, the role of children’s emotions and beliefs and how they influence recantations can be explored. As noted in the introduction, even if family members are forthcoming about their supportiveness, the child’s perception of support may be a better source of information in understanding the child’s decision whether to recant.

Third, about half of children referred to separation from their family. Intuitively, removal of the child from the home and placement in foster care provides a strong motivation for recantation (London et al., 2008). However, research has not borne this out: Malloy and colleagues (2016) found that children removed from their home were *less* likely to recant. This relation was no longer statistically significant when they controlled for maternal supportiveness, which suggests that the effects of removal on recantation depend on whether removal means separation from maternal influence. Removal from the home may have a double-sided effect: on the one hand the child misses their family, but on the other hand the child is less likely to be exposed to maternal pressure. In cases in which children have been removed and nevertheless recant, their removal is a highly salient reminder of the effects of their disclosure, and it is not surprising they mention this when recanting. Future research can examine the effects of removal in a more nuanced way, by examining interactions between the effects of removal and family supportiveness, and by considering the type of removal. It may be that removal increases the likelihood of recantation when children are placed with or have frequent contact with unsupportive family members.

### The Relative Frequency of Influences and Pressures

Children more often mentioned influences than overt pressures to change their story. This could reflect the fact that influences are in fact more common than overt pressure. Mothers and others who are uncertain about the truth of the allegations may be more likely to express their doubts in indirect ways, rather than directly confronting the child. Indeed, the pressures that children revealed to be emanating from the mother tended to be less direct, such as repeatedly asking the child if the allegations were true, rather than explicit statements of disbelief or explicit directions to recant. Furthermore, there are legal reasons for mothers to avoid overtly pressuring the child. Parents in dependency cases are often ordered not to discuss the allegations with the child (*In re E.G*., 2025). If they obey the admonition, the child is nevertheless likely to feel the influence of what they can observe, such as the mother’s sadness that the suspect is out of the home. If they disobey the admonition, and explicitly pressure the child, children may avoid mentioning pressures because of their awareness that this would undermine the persuasiveness of their recantation. For example, in *In re E.G.* (2025), the recanting 11-year-old “testified no one had promised her anything or pressured her into changing her account,” (p. 7), despite extensive evidence of skeptical questioning and suggestions by the mother and the maternal aunt.

The fact that children describe influences more often than pressures has implications for practice and future research. First, interviewers and legal professionals should take note of influences that may lead children to recant even when there is no evidence of overt pressure. When pressures are most effective, they not only lead the child to recant but also to deny that they were pressured to do so (Lyon & Dente, 2012). Hence, influences may be the only available evidence. Second, when conducting interviews with younger siblings, which are customarily undertaken to assess risk, interviewers could include questions about what they and the mother have said about the older sibling’s allegations, because younger siblings may be more forthcoming about pressures.

Third, developmental research could examine what children understand about pressures and how acknowledging those pressures affects their credibility. Younger recanters are likely to be more forthcoming about external pressures when they occur because they won’t recognize that an observer will discount their story if they have been told to deliver it (Wylie et al., 2022). Conversely, they are more likely to believe that a parent’s statement of disbelief is convincing evidence that the event did not occur, and they will be motivated to tell the interviewer about it. More generally, developmental research can explore age differences in children’s ability to recall influences and pressures, which is likely related to their ability to recall conversations, an active area of research (Lawson & London, 2017; Stolzenberg et al., 2018).

### Limitations and Future Directions

The cases examined in this sample were limited to recantations of abuse during a specific time point in the disclosure and investigation of sexual abuse: a formal interview with a forensic interviewer who was assessing whether protective services should limit the custodial rights of the child’s caregiver and whether criminal charges should be filed against the suspect. Of course, recantations occur in many other contexts. Children may recant informally; for example, they sometimes recant to their mother or other non-professionals. Children may recant at other times: both before or after the formal interview. Their case might be heard in dependency proceedings (as was true in almost all of our cases), but also in criminal or family court, and they could recant before, during, or after their testimony. All of our cases involved familial suspects, but children may recant against strangers or familiar suspects with no ongoing access to the child.

As with most observational research examining child maltreatment, another limitation is ground truth. It is possible that children’s recantations were true and that their initial allegations were false. Although 90% of our cases were substantiated by social services and a dependency petition was filed, this does not guarantee that the allegations were true. Conversely, the three cases in which DCFS failed to substantiate the allegations (69, 129, 321) are not necessarily untrue, particularly since the recantation underlay DCFS’ failure to substantiate. In the case of 12-year old Addison (69), the child disclosed to a teacher and to the police that her mother’s boyfriend had repeatedly touched her “private parts” (which she defined as her chest and her vaginal area) and tried to kiss her on the mouth when she was alone with him, acts which she said he called their “little secrets.” In the forensic interview, she acknowledged her disclosures to the teacher and the police, and also disclosed that she reaffirmed the abuse when her mother questioned her after telling her the suspect had gone to jail. She recanted at the interview, explaining that she was angry at the suspect for breaking her radio. The police dropped their investigation and DCFS found the allegations “inconclusive.” 13-year-old Daniela (129) disclosed to her mother that she had sex with a man whose name began with the letter “R.” The mother recognized that she was referring to the stepfather, but he denied it when she confronted him. The child then repeated the allegation to a friend at school, and at her urging, the vice principal. When questioned by the police, she ultimately recanted, but then realleged abuse when questioned by a social worker, complaining that the police were being aggressive and that no one was listening to her. She recanted to the CAC interviewer, and did not provide a clear reason for alleging abuse. The police dropped their investigation and DCFS found the allegations “inconclusive.” 10-year-old Sofia (321) disclosed to her sisters, her mother, a friend, a social worker and the police that when her mother was at work her mother’s boyfriend had pulled her out of her bed (which was next to his) and touched her “private parts” (which she defined as her chest and her vaginal area), though she was unsure if she was dreaming. Her mother took her to a fortune teller who advised that it was just a dream. In the forensic interview she stated that “it felt real” but “I’m pretty sure it’s a dream because he’s like ‘I don’t, I don’t remember touching you’ and he said that he loves me.” The police closed the investigation and DCFS found the allegations “unfounded.”

One particularly important topic for future research is to examine cases in which children recant at an early stage of the investigation, leading social services to close the case. Because the major source of our cases was dependency court, we had only three cases of this type in our sample, yet they are quite common (Leach et al., 2017; Lippert et al., 2009), and the possibility that they are true allegations deserves closer scrutiny (Everson & Boat, 1989).

In considering whether the initial allegations were true or not, it is important to recognize that children’s disclosure of influences and pressures to recant does not itself constitute evidence of abuse. Children making false allegations are surely also often subject to pressures to change their stories. Influences and pressures do not increase the likelihood of abuse, but to the extent that they lead both abused and non-abused children to recant, decrease the likelihood that recantations are proof that abuse did not occur. They are thus analogous to proof of influences and pressures on the child to allege abuse, which can occur in both true and false allegations, but nevertheless can reduce the evidentiary value of a disclosure (Lyon et al., 2026).

Future research can create a clearer picture of influences and pressures on abused children to recant. First, future research can examine the time course of recantations, identifying them along the course of initial discovery and investigation. Second, research can move beyond qualitative assessment and develop an objective and reliable coding scheme for identifying influences and pressures. This will enable future research to examine the relation between influences and pressures and characteristics of the child, the case, and the kinds of questions that were asked. Examining the kinds of questions that most effectively elicit influences and pressures will be of particular interest to interviewers who question recanting children (Chase & Stiles, 2015).

Understanding potential pressures and influences faced by children following allegations of abuse not only helps investigators in identifying cases in which recantation might be anticipated but also in planning an appropriate interview strategy for questioning children who have recanted or at high risk of recanting their allegations. Despite recognition that recantation interviews may require a different approach, current guidelines offer little guidance on how interview strategies can be adapted (Lamb et al., 2018). This study provides a first step toward understanding how best to question children who recant allegations of sexual abuse. Most children were forthcoming about some influences and pressures, suggesting that questions about the reactions of disclosure recipients and about the child’s feelings concerning the disclosure and its aftermath may elicit details that help investigators assess the motivations behind the child’s recantation. Future research examining the effectiveness of these prompts using a reliable, quantitative coding scheme will build the evidence base required for making concrete, practice-oriented recommendations.

### Conclusion

Thematic analysis of 53 forensic interviews with 4- to 15-year-old children who recanted allegations of intrafamilial sexual abuse revealed that almost 90% of children mentioned influences or pressures to recant. Children most frequently disclosed influences related to the suspect and family and acknowledged direct pressures less often in comparison. Together with research demonstrating the role of familial influences in recantation, the findings have important implications for the way forensic interviewers question children who recant allegations of sexual abuse.

## Data Availability

The datasets analyzed for the current study are not publicly available due to ethical considerations (anonymity of children).*
